# *Anditalea andensis* ANESC-S^T^ - An Alkaliphilic Halotolerant Bacterium Capable of Electricity Generation under Alkaline-Saline Conditions

**DOI:** 10.1371/journal.pone.0132766

**Published:** 2015-07-14

**Authors:** Wei Shi, Victor Bochuan Wang, Cui-E Zhao, Qichun Zhang, Say Chye Joachim Loo, Liang Yang, Chenjie Xu

**Affiliations:** 1 School of Chemical and Biomedical Engineering, Nanyang Technological University, Singapore, Singapore; 2 Singapore Centre on Environmental Life Sciences Engineering, Nanyang Technological University, Singapore, 637551, Singapore; 3 School of Materials Science and Engineering, Nanyang Technological University, Singapore, 639798, Singapore; 4 School of Biological Sciences, Nanyang Technological University, Singapore, 637551, Singapore; 5 Key Laboratory of Flexible Electronics & Institute of Advanced Materials, National Jiangsu Synergistic Innovation Center for Advanced Materials, Nanjing Tech University, 30 South Puzhu Road, Nanjing, 211816, P. R. China; Institute of Genetics and Developmental Biology, Chinese Academy of Sciences, CHINA

## Abstract

A great challenge in wastewater bioremediation is the sustained activity of viable microorganisms, which can contribute to the breakdown of waste contaminants, especially in alkaline pH conditions. Identification of extremophiles with bioremediation capability can improve the efficiency of wastewater treatment. Here, we report the discovery of an electrochemically active alkaliphilic halotolerant bacterium, *Anditalea andensis* ANESC-S^T^ (=CICC10485^T^=NCCB 100412^T^), which is capable of generating bioelectricity in alkaline–saline conditions. *A*. *andensis* ANESC-S^T^ was shown to grow in alkaline conditions between pH 7.0–11.0 and also under high salt condition (up to 4 wt% NaCl). Electrical output was further demonstrated in microbial fuel cells (MFCs) with an average current density of ~0.5 µA/cm^2^, even under the harsh condition of 4 wt% NaCl and pH 9.0. Subsequent introduction of secreted extracellular metabolites into MFCs inoculated with *Escherichia coli* or *Pseudomonas aeruginosa* yielded enhanced electrical output. The ability of *A*. *andensis* ANESC-S^T^ to generate energy under alkaline–saline conditions points towards a solution for bioelectricity recovery from alkaline–saline wastewater. This is the first report of *A*.*andensis* ANESC-S^T^ producing bioelectricity at high salt concentration and pH.

## Introduction

Microbial fuel cell (MFC) is an emerging technology which employs exoelectrogens to concurrently bioremediate and produce energy from wastewater [1–3]. It holds promise in reducing energy consumption in wastewater treatment, which costs 3% of electricity in US annually [4]. Research into exoelectrogens has blossomed in the past decade with the discovery of >50 microbial species, such as *Shewanella oneidensis* and *Geobacter sulfurreducens*, which possess inherent bioelectricity generation capabilities [5–7]. In MFCs employing such species, organic substrates are oxidized in the anaerobic chambers and liberated electrons are transferred to extracellular electrodes through a variety of charge transfer mechanisms, such as membrane associated cytochromes, secretion of soluble redox mediators and physical appendages such as conductive pili [8–11]. Unfortunately, most of these exoelectrogens are only functional under neutral pH and low salt (<3.3%) conditions. This constitutes a bottleneck for MFCs to be employed in processing wastewater with high salt and/or highly alkaline conditions [12,13].

**Fig 1:**
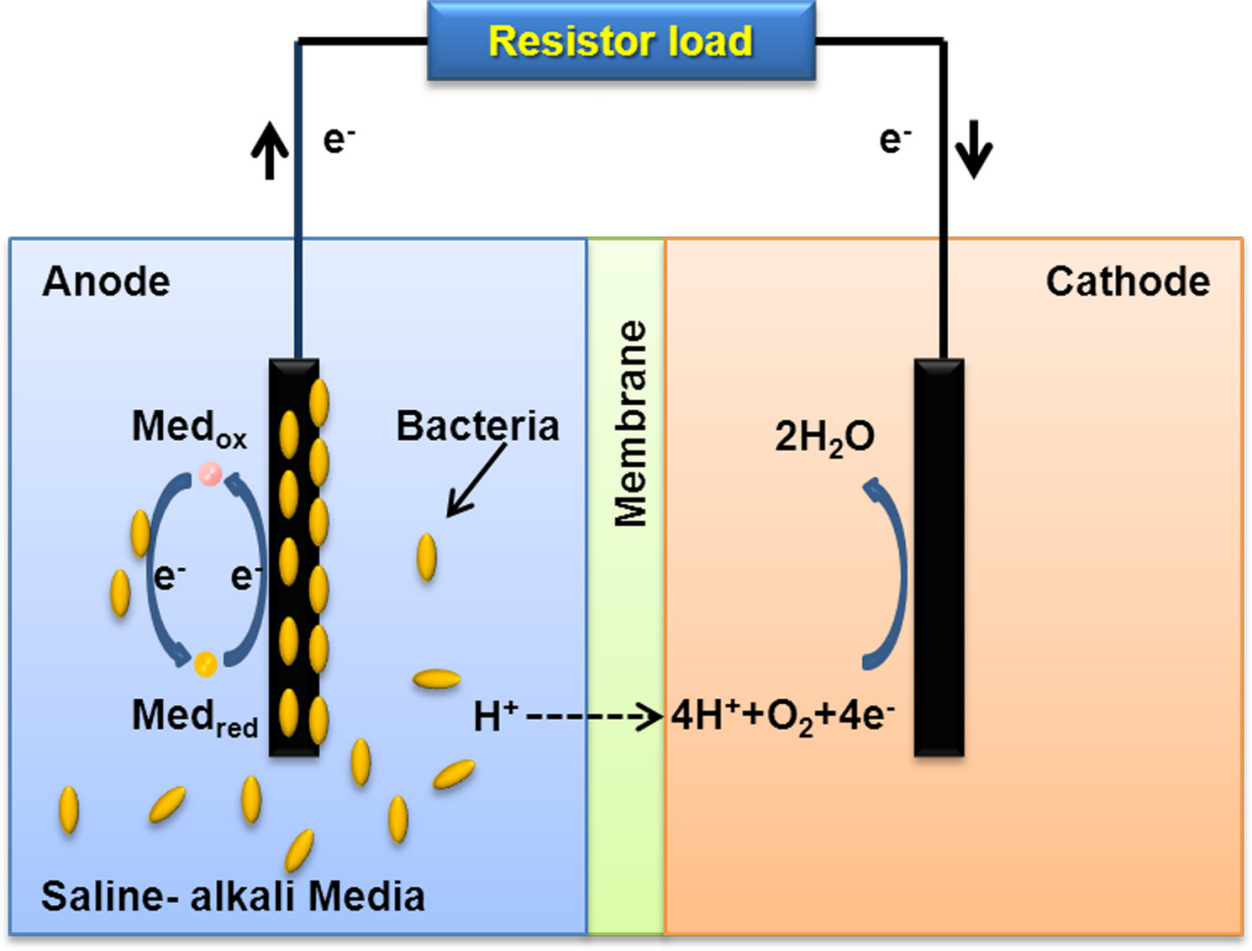
Schematic illustration of two-chambered MFC for bioelectricity generation.

Previously, we isolated a salt- and alkaline-tolerant bacterium, *Anditalea andensis* ANESC-S^T^, from extremely soda saline-alkali soil[14]. This strain is able to grow in alkaline conditions (between pH 7.0–11.0) and under high salt concentrations (up to 4 wt% NaCl). Further, it belongs to the phylum ‘Bacteroidetes’, of which most members are important heterotrophs for recycling organic carbon in freshwater and marine habitats [15]. In this study, two-chamber MFCs (Fig 1 and S1 Fig) were utilized to demonstrate the bioelectricity generation capabilities of this species under high salt and/or highly alkaline conditions. An average current density of ~0.5 μA/cm^2^ was maintained for ~ 450 hours. Finally, we revealed the extracellular charge transfer mechanism employed by this species and show its bioelectricity generating capability in seawater. This demonstration points towards possible utilization of this species for simultaneous bioremediation and bioelectricity generation in harsh conditions, which are prevalent in wastewater treatment.

## Materials and Methods

### Bacterial strains and culture conditions

*A*. *andensis* ANESC-S^T^ was obtained from China Center of Industries Culture Collection (CICC) (Beijing, China). *Escherichia coli* DH5α, and *Pseudomonas aeruginosa lasIrhlI* mutant were obtained from American Type Culture Collection (ATCC). *E*. *coli* DH5α and *P*. *aeruginosa lasIrhlI* were cultured in LB broth at 37^°^C. *A*. *andensis* ANESC-S^T^ was cultured in LB agar (pH 7.0, 1% NaCl) at 30^°^C. All cells were harvested by centrifugation (8000 rpm at 4^°^C). Cells were then re-suspended in the anolyte for use in MFCs. *A*. *andensis* ANESC-S^T^ was grown in M9 containing 10 mM L-Arabinose and 5% LB for use in cyclic voltammetry.

### Viability of *A*. *andensis* ANESC-S^T^in alkaline–saline conditions

*A*. *andensis* ANESC-S^T^ was grown in LB broth until OD_600_ = 0.6, and further diluted 10^0^, 10^−1^, 10^−2^, 10^−3^, 10^−4^ times with sterile physiological saline. 4 μL of each dilution were inoculated to agar plates with different alkaline–saline conditions. The plates were then incubated at 30°C for 48 hours and their growth was monitored every 12 hours.

### MFC construction

All media for MFCs were sterilized by autoclaving. MFCs were dual chamber, U-tube devices constructed as reported previously (S1 Fig) [16]. The anode and cathode chambers were made of two 90° 28/15 ball-to-plain-end and socket-to-plain-end glass adapters (17 mm O. D. × 1.8 mm wall thickness) (VWR) separated from each other by a circular piece of Nafion N117 (Ion Power, USA) proton exchange membrane (PEM). The glass tubes were sealed against the PEM with high-vacuum silicone grease and held in place with a 28/15 stainless steel pinch clamp (VWR). Carbon felt electrodes (3.18 mm thickness) (VWR) were cut to 2 cm × 5 cm dimensions (width × length) and connected at one end with titanium wire (0.25 mm diameter) (Sigma-Aldrich Corp.) with nylon screws and nuts (Small Parts, Inc.A). Prior to MFC operation, the devices were filled with ultrapure water and autoclaved to sterilize the devices and internal components. After sterilization and decanting off the ultrapure water, the anode and cathode chambers were each filled with the respective sterile medium with the required pH and salt concentrations. The required volume of live cell culture solution was then inoculated into the anode chamber. The final total volume of solution in each of the anode and cathode chambers was maintained at 20 mL. The anode chamber was sealed with a serrated silicone septum (18 mm O. D.) (Sigma-Aldrich) through which the titanium wire was threaded, while the cathode chamber was loosely capped with an inverted glass scintillation vial. The cathode electrodes were only partly submerged in the catholyte to allow for an ‘air-wicking’ aerobic configuration. The electrodes were then connected to 1 kΩ resistors and voltage measurements were recorded at a rate of 1 point per 5 min using an eDAQ e-corder data acquisition system equipped with Chart software (Bronjo Medi, Singapore). Data collection started immediately after inoculation of the devices. The MFCs were operated inside an incubator at 30°C. All runs were conducted in triplicates and data was further averaged. Voltage readings are collected as raw data and further converted to current density for presentation according to the following equation.

I=V/R

where I is the current in amperes (A), V is the potential difference in volts (V) and R is the resistance in ohms (Ω). Current density is obtained by dividing the equation above by the geometrical surface area of the electrode (20 cm^2^).

**The polarization curves** were obtained by using linear sweep voltammetry on a CHI660E electrochemical workstation (CH instruments, Chenhua, China) with a three-electrode system. The scan rate was 1 mV s^-1^. The current was calculated using *I = V/R*, and the power was obtained as *P = IV*. Power density was normalized to the project area of the anode area.

### Extraction of extracellular metabolites

*A*. *andensis* ANESC-S^T^ was grown on agar plates (pH 9.0, 1 wt% NaCl for 72 hours at 30^°^C). Cells were scraped from 10 agar plates with a sterile spatula and washed with 50 mL deionized (DI) water (Millipore). They were subsequently scraped off and dissolved in sterile water for 1 hour. The suspension was centrifuged (10000 *g*) to remove planktonic bacteria, and the supernatant was filtered through 0.22 μm membrane filters (Pall Corp.). The resultant solution was then freeze-dried (Labconco freezone 4.5, USA).

### Cyclic Voltammetry (CV)

This measurement was performed using an electrochemical analyzer (CHI604E, CH Instruments Inc.) with a standard three electrode system. The graphite felt electrode and the platinum wire functioned as the working and the counter electrode, respectively. The reference electrode was Ag/AgCl and was inserted into the anode compartment. The parameters for CV characterization were: equilibrium time: 2 seconds (at −0.7 V), scan rate: 5 mV/s. All scans were performed from -0.7 to 0.2 V. CV measurements with cell-free spent medium and non-inoculated control medium were performed using a pre-sterilized electrode in the same reactor. Chronoamperometric curves were obtained at 0.497 V vs. Ag/AgCl after the carbon felt electrodes were immersed for 30 minutes in the M9 media containing 5% LB and 10mM L-Arabinose at various pHs.

## Results and Discussion

**Fig 2:**
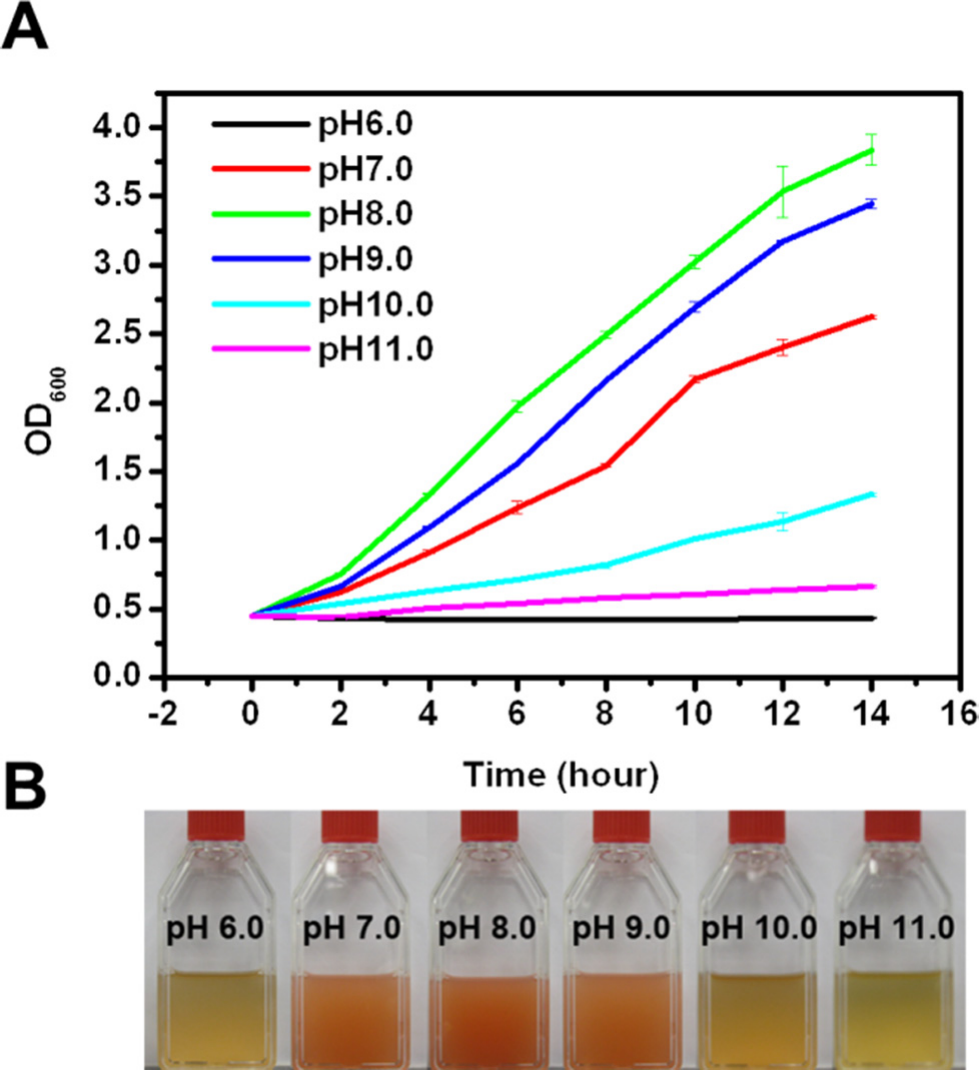
Analysis of *A*. *andensis* ANESC-S^T^ resilience to different pH conditions. (A) Growth curves of *A*. *andensis* ANESC-S^T^ in LB broth with varied pH conditions (Initial OD_600_ ≈ 0.4). (B) Cultures of *A*. *andensis* ANESC-S^T^ after 14 hours in LB broth with varied pH conditions (Initial OD_600_ ≈ 0.4).

The ability of *A*. *andensis* ANESC-S^T^ to grow at high pH conditions was characterized by examining its viability in both Luria-Bertani (LB) agar (1% NaCl) (S2 Fig) and LB broth (1% NaCl) (Fig 2A and 2B), which are common growth media. *A*. *andensis* ANESC-S^T^ was cultured overnight (OD_600_ ≈ 0.6), subsequently diluted in a series (10^0^ to 10^−4^ diluted cultures) and plated onto LB agar plates with different pH conditions (pH 7.0–11.0). After 48 hours, the plates exhibited different depths of orange, which corresponds to the varying bacterial concentration. *A*. *andensis* ANESC-S^T^ showed proliferation between pH 7.0–11.0, while its growth was inhibited at pH 6.0 (S2 Fig). Specifically, optimal growth was observed at pH 8.0 and 9.0, which suggested efficient nutrient absorption at these pH values. Further, we examined the growth of *A*. *andensis* ANESC-S^T^ in LB broth at different pH conditions. Consistent with the above observation, this bacterium demonstrated optimal growth at pH 8.0 and 9.0 (Fig 2A and 2B).

**Fig 3:**
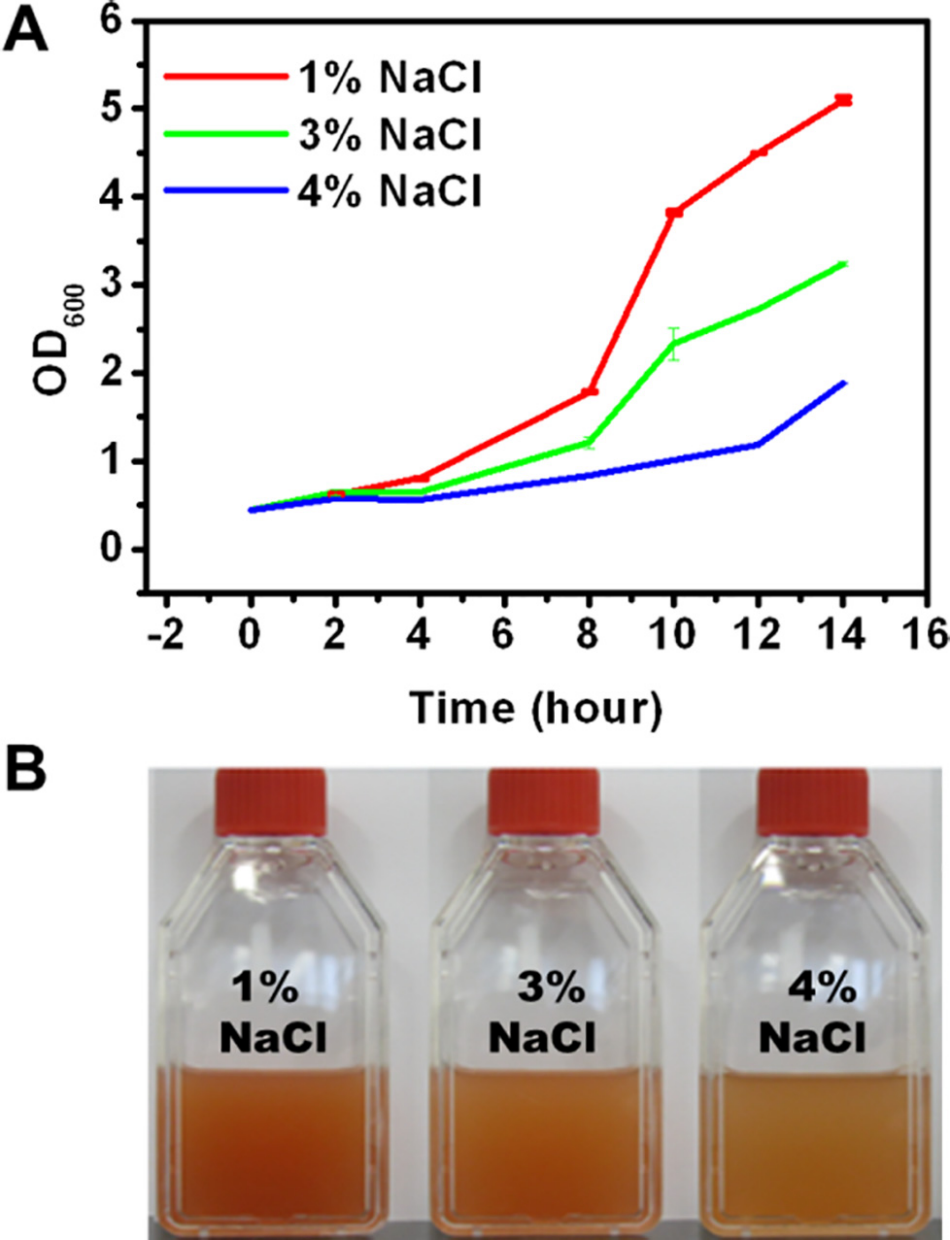
Analysis of *A*. *andensis* ANESC-S^T^ resilience to different salt concentrations. (A) Growth curves of *A*. *andensis* ANESC-S^T^ in LB broth with varied NaCl concentrations (Initial OD_600_ ≈ 0.4). (B) *A*. *andensis* ANESC-S^T^ was inoculated and cultured for 14 hours in LB broth with varied NaCl concentrations (Initial OD_600_ ≈ 0.4).

Next, the viability of *A*. *andensis* ANESC-S^T^ under various salt concentrations was examined. Specifically, LB agar plates (pH 9.0) with different NaCl concentrations (1%, 3% and 4%) were prepared. *A*. *andensis* ANESC-S^T^ was cultured overnight (OD_600_ ≈ 0.6) and inoculated under different seeding concentrations (10^0^ to 10^−4^ diluted cultures). After 48 hours, growth was observed in all conditions (S3 Fig). This was confirmed by its growth in LB broth (pH 9.0) with different NaCl concentrations (Fig 3A and 3B). Growth curves of both species corroborated the observations (Fig 3A).

**Fig 4:**
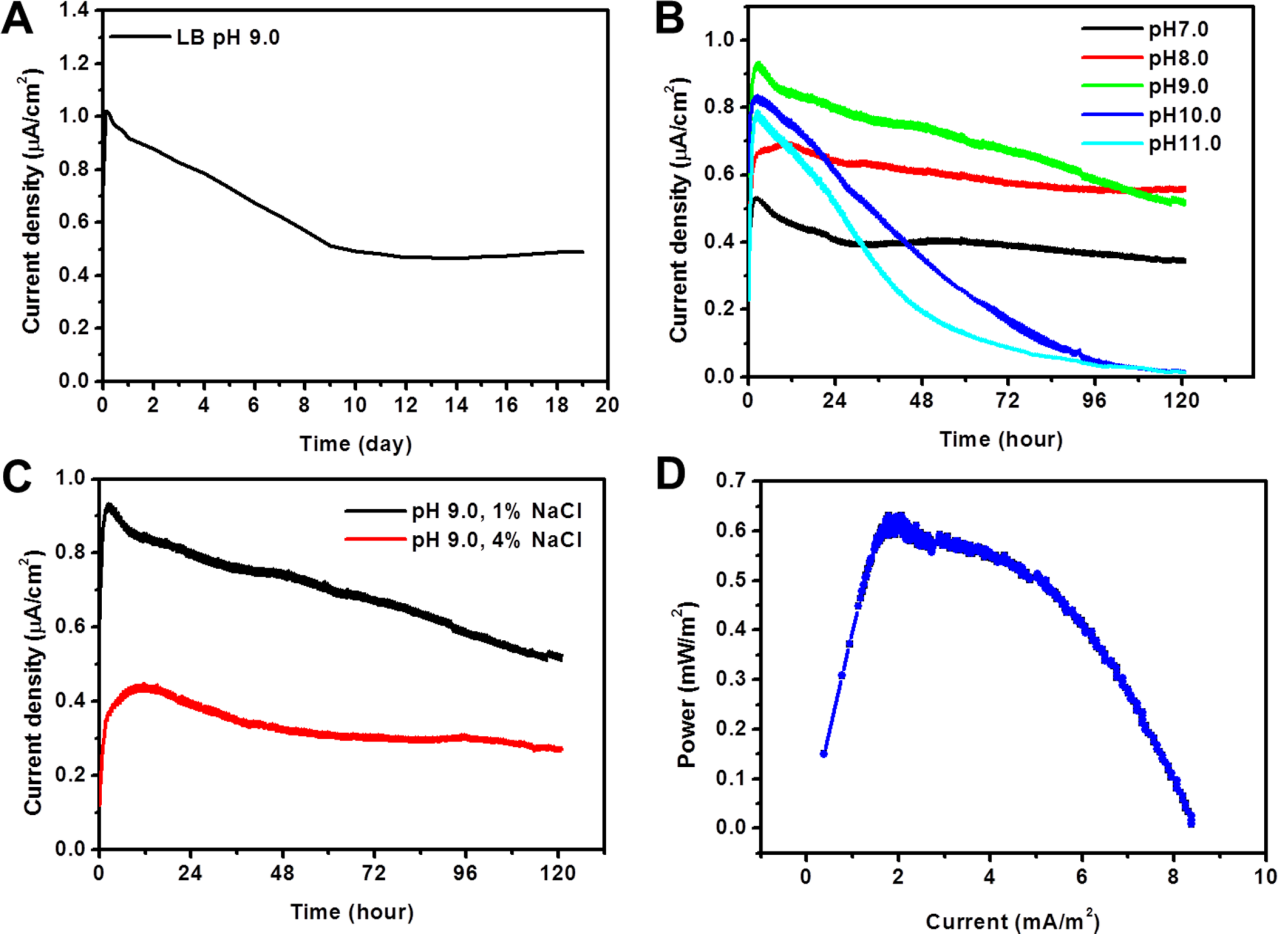
Bioelectricity generation by MFCs inoculated with *A*. *andensis* ANESC-S^T^ operated under different pH and salt concentrations. (A) Current density vs. time plot of *A*. *andensis* ANESC-S^T^ in LB broth (pH = 9.0, 1% NaCl). (B) Current density vs. time plot of *A*. *andensis* ANESC-S^T^ in LB broth with pH 7.0, 8.0, 9.0, 10.0, and 11.0. (C) Current density vs. time plot of *A*. *andensis* ANESC-S^T^ in LB broth with 1% and 4% NaCl. Data represents an average of triplicates. (D) Power density curve of the MFC inoculated with *A*. *andensis* ANESC-S^T^ in LB broth (pH 9 and 1% NaCl).

*A*. *andensis* ANESC-S^T^ has been previously shown to belong to the phylum ‘Bacteroidetes’, which contains exoelectrogens such as *Bacteroides sp*. W7 [17,18]. Hence, we hypothesized that *A*. *andensis* ANESC-S^T^ might generate bioelectricity as well. Two chamber MFCs (S1 Fig) were thus employed to investigate the bioelectricity generation capability of *A*. *andensis* ANESC-S^T^. Specifically, MFCs were inoculated with *A*. *andensis* ANESC-S^T^ grown in LB broth (pH = 9.0, 1% NaCl, OD_600_ ≈ 2.0) and produced a stable electrical output with an average current density of ~0.5 μA/cm^2^ for ~450 hours (Fig 4A).

Further electrical characterization using MFCs was performed to fully study the bioelectricity generation capability of this strain under different pH and salt concentrations (Fid. 4B, C). LB medium was titrated to specific pH values (i.e. 7.0, 8.0, 9.0, 10.0, and 11.0) before being inoculated with *A*. *andensis* ANESC-S^T^. The strain was allowed to grow to OD_600_ ≈ 2.0 before being transferred to the MFC anode chambers. Stable current density ranging from ~0.35 to ~0.90 μA/cm^2^ could be observed under pH conditions of 7.0, 8.0, and 9.0 over 120 hours (Fig 4B).

Given the small current densities observed, one of the concerns is whether a significant amount of current could originate from redox chemistry from the complex LB medium. Thus, the chronoamperometric curves were plotted for fresh M9 media containing 5% LB at four different pH values (S4 Fig). At pH 7.0 and 8.0, the current densities were the lowest at ~0.09 μA/cm^2^. The current density increased to 0.12 μA/cm^2^ at pH 9.0, and 0.18 μA/cm^2^ at pH 10.0. This result suggests that the current observed in MFCs inoculated with *A*. *andensis* ANESC-S^T^ (Fig 4B) came partially from redox chemistry from the complex LB medium. Further, this background current was pH dependent. Nevertheless, the current densities showed by the bacterium-free medium at pH 7.0, 8.0 and 9.0 were still much lower than the values recorded in MFCs (0.09, 0.09, and 0.12 μA/cm^2^ vs. 0.4, 0.6, and 0.7 μA/cm^2^ respectively). At pH 10.0, despite observing the highest current density from the media, the MFCs failed to provide a stable output. In other words, the current output observed in MFCs was mainly due to the alkaliphilic and exoelectrogenic *A*. *andensis* ANESC-S^T^.

Later, the bioelectricity generation capability of *A*. *andensis* ANESC-S^T^ in LB medium with different NaCl concentrations (1% and 4%) was examined while maintaining the optimum pH at 9.0. LB in the experiments normally contains 1% NaCl or 170mM NaCl, which is sufficient for providing conductivity and maintaining constant ionic strength. The ionic conductivity of LB solution at 9 is 26.125±0.075 ms/cm. The increase of NaCl concentration (pH = 9) from 1% to 4% further improved the ionic conductivity from 26.125±0.075 ms/cm to 74.6±0.2 ms/cm.

Although *A*. *andensis* ANESC-S^T^ exhibited better electrical performance in 1% NaCl, it is noteworthy to mention that the electrical output at 4% NaCl was still significant at ~50% of the value of the devices maintained at 1% NaCl (Fig 4C). The power density curve is shown in Fig 4D, where the maximum power density is about 0.6 mW/m^2^ at a current density of 2.0 mA/m^2^.

Taken together, *A*. *andensis* ANESC-S^T^—based MFCs show promise for producing bioelectricity in salt and alkaline environments. Although the current density is still low, further optimization to the device architecture or internal components can be performed for sustainable and enhanced bioelectricity generation [19].

**Fig 5:**
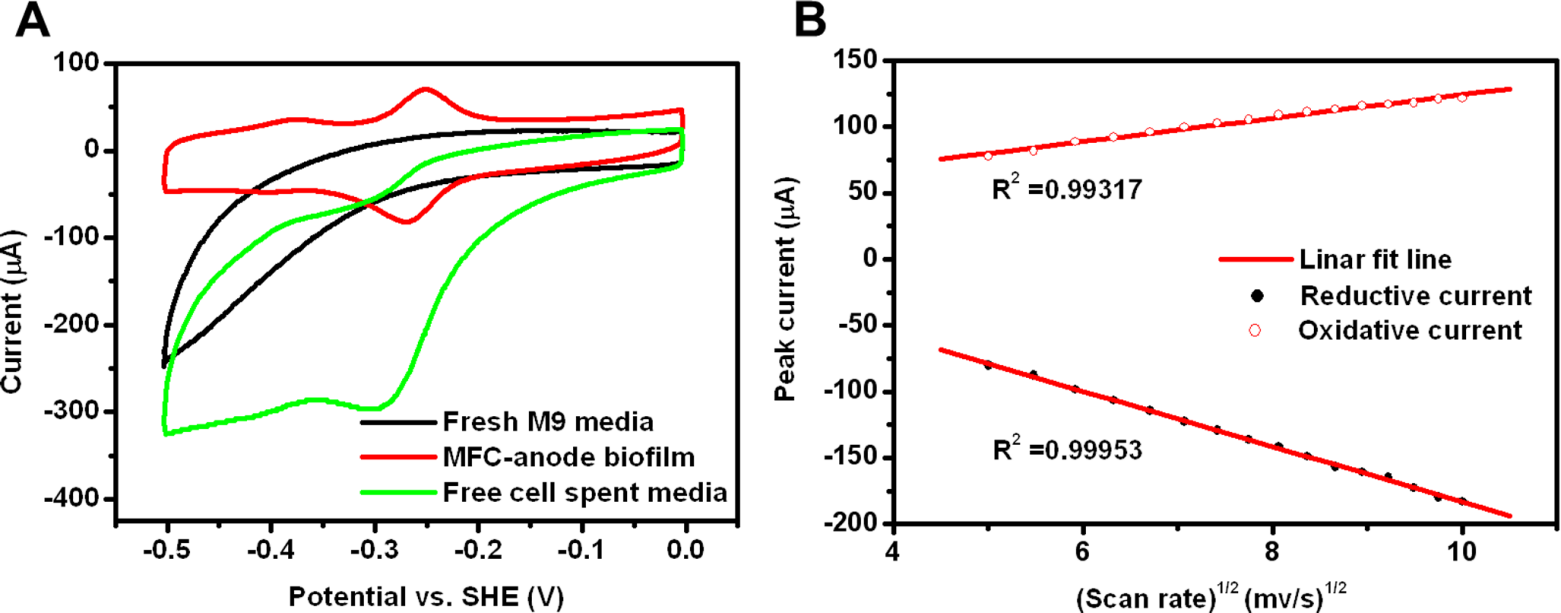
CV analysis of *A*. *andensis* ANESC-S^T^ in MFCs. (A) CV traces of MFCs inoculated with fresh M9 media, *A*. *andensis* ANESC-S^T^ and free cell spent media of *A*. *andensis* ANESC-S^T^. The working electrode is carbon felt, counter electrode is Pt wire and the reference electrode is Ag/AgCl. Scan rate is 5 mV/s. Voltammograms were acquired in the presence of 10 mM L-Arabinose. (B) Plot of peak current vs. scan rate of the CV traces. All experiments were performed in triplicates.

Next, the electron transfer between *A*. *andensis* ANESC-S^T^ and electrode surfaces in MFCs was evaluated by cyclic voltammetry (CV) [20]. CV was conducted on the fresh media, the cell-free spent media and *A*. *andensis* ANESC-S^T^ culture. The CV of *A*. *andensis* ANESC-S^T^ indicates that a pair of major redox peaks was observed at -0.253 V and -0.269 V (vs. SHE), which implies that the catalytic reaction is quasi-reversible (Fig 5A, red trace). The control sample using non-inoculated electrode with fresh media showed no catalytic current (Fig 5A, black trace), which indicates that the electrochemical active compound was excreted by *A*. *andensis* ANESC-S^T^ during MFC operation. However, the cell-free spent media acquired from the anode chambers of operated MFCs (Fig 5A, green trace) produced catalytic current, which supported the hypothesis that *A*. *andensis* ANESC-S^T^ secreted compounds responsible for the electron transfer from the cell surface to the electrode of the MFCs [21]. It should further be pointed out that peak potentials from *A*. *andensis* ANESC-S^T^ MFCs were similar to those from the free cell spent media. Our data is in accordance with similar work which has previously been undertaken to demonstrate the relationship of secreted metabolites in electron transfer, for example, flavins as electron shuttles in *S*. *oneidensis* [11] and pyocyanin reprising the same role in *P*. *aeruginosa* [22]. This suggests that the electrochemical active compounds found to be secreted from *A*. *andensis* ANESC-S^T^ were participating in extracellular electron transfer (EET) in the MFCs.

To further elucidate the EET mechanism, peak currents corresponding to various scan speeds were analyzed (Fig 5B), which showed peak currents increasing linearly with the square root of scan rate. This suggests that redox compounds were not adsorbed on the electrode surface and the EET mechanism of *A*. *andensis* ANESC-S^T^ in MFCs depends on the diffusive redox process [23]. This suggests that the electrochemical active compounds excreted by *A*. *andensis* ANESC-S^T^ were possibly involved in bioelectricity generation at an anode in the absence of exogenous electrochemical active compounds. Further work is undergoing to reveal the specific types of electrochemical active compounds.

**Fig 6:**
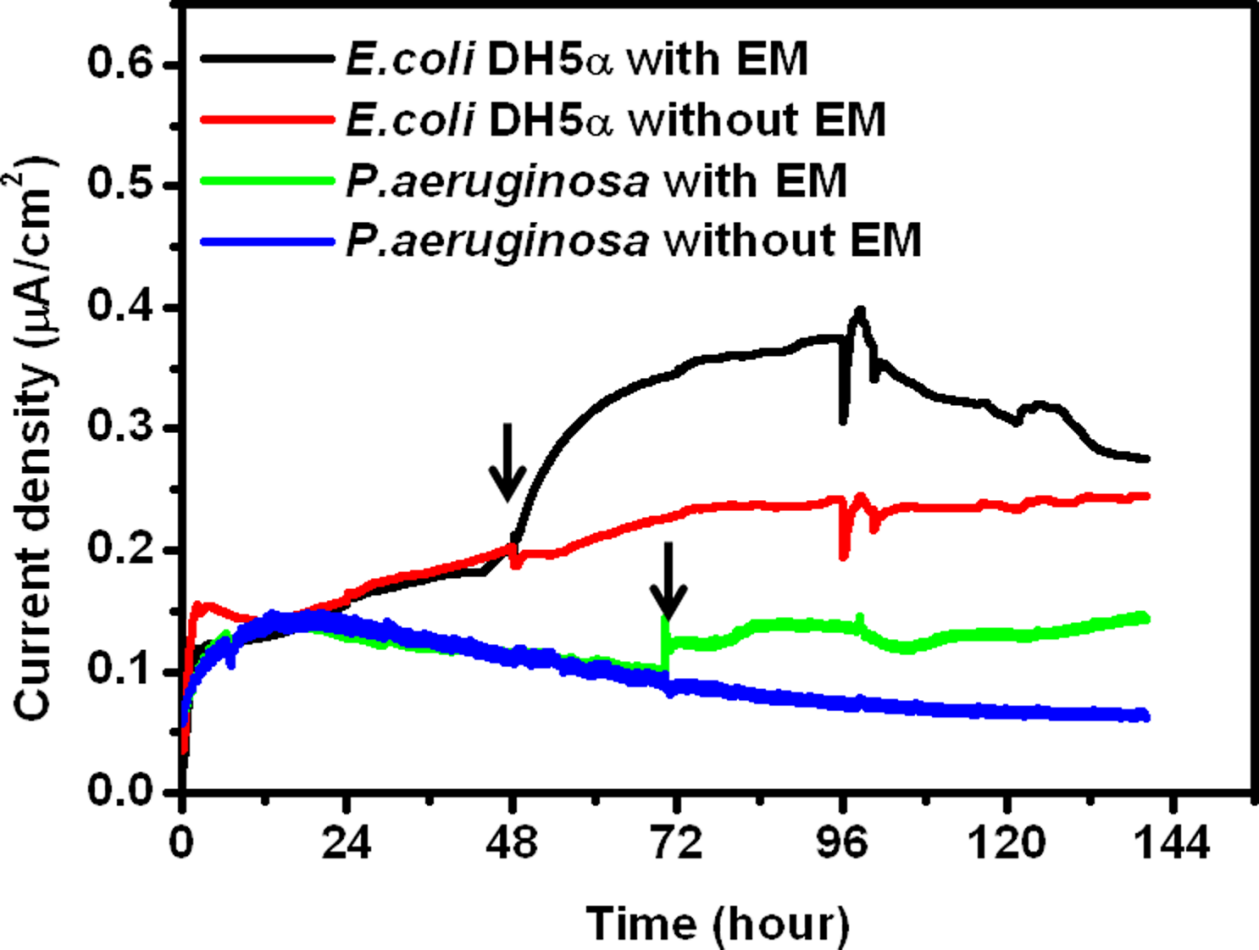
Current density vs. time plots of *E*. *coli* DH5α and *P*. *aeruginosa lasIrhlI* mutant – based MFCs. EM stands for extracellular metabolites of *A*. *andensis* ANESC-S^T^ (2.5 mg/mL). Arrows indicate introduction of EM. Data represents an average of triplicates.

It is well established that the electrochemical active compounds secreted by one species can improve the EET rates of other species [10]. In this light, we examined the influences of *A*. *andensis* ANESC-S^T^’ extracellular metabolites to bioelectricity generation of MFCs containing either *E*. *coli* DH5α or phenazine defective *P*. *aeruginosa lasIrhlI* mutant (Fig 6) [24]. Isolated extracellular metabolites secreted by *A*. *andensis* ANESC-S^T^ were introduced to each MFC only after current densities saturated. Bioelectricity generation from *E*. *coli* DH5α MFCs improved from ~0.15 μA/cm^2^ to ~0.30 μA/cm^2^ upon addition of extracellular metabolites (Fig 6, red versus black trace). It should be mentioned that after addition of extracellular metabolites, there was only a minor change in the number of *E*. *coli *DH5α in MFCs (i.e. 48 hours: 5.55±0.31 × 10^7^ cells/mL, 72 hours without addition: 5.7±0.5 × 10^7^ cells/mL, 72 hours with addition: 5.9±0.9 × 10^7^ cells/mL). The insignificant change in cell numbers suggests that the increase was due to enhanced EET, brought by the introduction of extracellular metabolites of *A*. *andensis* ANESC-S^T^. A similar phenomenon was observed in the *P*. *aeruginosa lasIrhlI* mutant—inoculated MFCs. The addition of extracellular metabolites after 72 hours increased the current density from ~0.08 μA/cm^2^ to ~0.12 μA/cm^2^ (Fig 6, blue versus green trace). These collective analyses demonstrate that the extracellular metabolites of *A*. *andensis* ANESC-S^T^ contain the electrochemical active compounds, which facilitate EET in MFCs.

**Fig 7:**
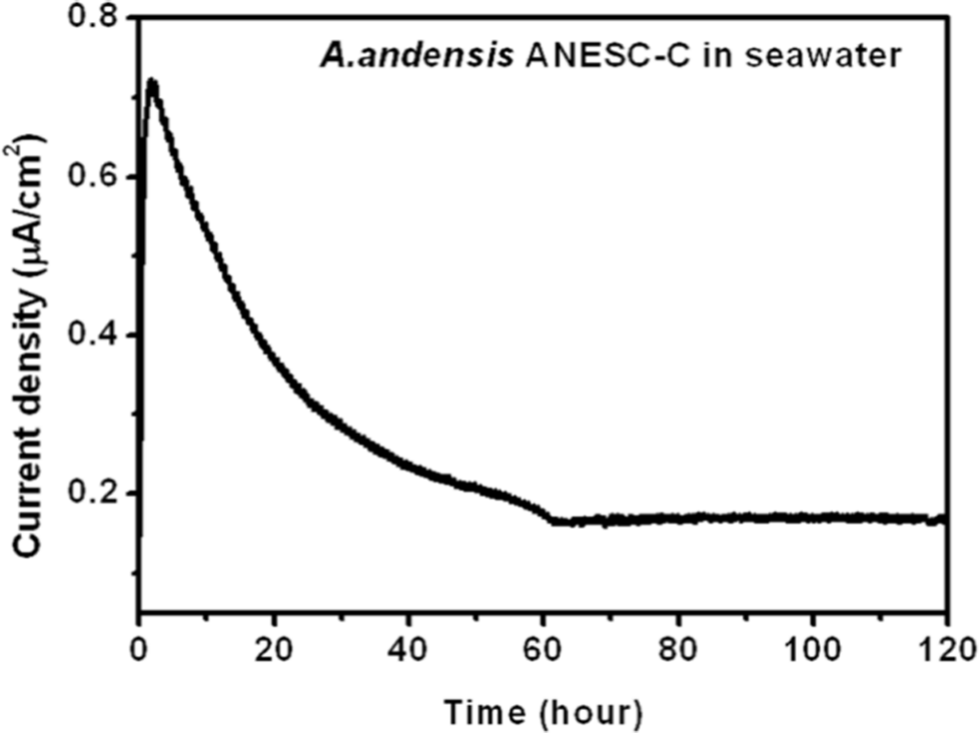
Current density vs. time graph of *A*. *andensis* ANESC-S^T^ in seawater fed MFCs. Data represents an average of triplicates.

Given the bioelectricity generation of *A*. *andensis* ANESC-S^T^ at alkaline–saline conditions, we suspect that it would find applications in conditions such as biodiesel production [25], paper and pulp mill, alkaline factory [26], and eutrophicated seawater processing, where normal bacterial species are unable to survive because of the alkaline and/or hypersaline environment. As proof-of-concept, *A*. *andensis* ANESC-S^T^ was introduced to MFCs containing seawater collected from the shoreline of Singapore. The current density showed a peak of ~0.75 μA/cm^2^, but gradually decreased and stabilized at ~0.2 μA/cm^2^ for at least 120 hours (Fig 7). The decline of bioelectricity production is attributed to the lower content of available nutrients of the seawater as the MFCs were operated in a batch fed mode.

## Conclusion

In this study, an alkaliphilic halotolerant bacterium, *A*. *andensis* ANESC-S^T^, was identified and characterized as an exoelectrogen. MFCs inoculated with this strain were shown to produce sustainable bioelectricity in alkaline–saline conditions with an average current density of ~0.5 μA/cm^2^. The current density is relatively low which might be attributed to high internal resistances within the devices that impede charge movement [27–30], because current densities from such adopted configuration typically fall in the range of μA [31–32]. In the future, the low current densities can be improved through various forms of optimization, such as employing different device architectures[33], apparatus components[34] or electrode/microbe interface engineering[29,30]. The electrochemical activity was traced to exogenously release soluble redox-active mediators and these compounds were found to enhance EET in MFCs containing other types of bacteria, which are commonly less electrochemically active. In view of these analyses, *A*. *andensis* ANESC-S^T^ might be employed as an alternative strategy for processing eutrophicated seawater.

## Supporting Information

S1 Fig Image of an *A*. *andensis* ANESC-S inoculated MFC. (TIF)

S2 Fig Analysis of *A*. *andensis* ANESC-S^T^ resilience to different pH conditions. Agar plates of *A*. *andensis* ANESC-S^T^ cultured for 48 hours under varied pH conditions with different seeding densities (Initial OD_600_ ≈ 0.6 for 10^0^ to 10^−4^ diluted cultures).
(TIF)

S3 Fig Analysis of *A*. *andensis* ANESC-S^T^ resilience to different salt concentrations. *A*. *andensis* ANESC-S^T^ was inoculated and cultured for 48 hours in LB agar with varied NaCl concentrations (1%, 3%, and 4%) under different seeding densities (10^0^ to 10^−4^ diluted cultures).
(TIF)

S4 Fig Chronoamperometric measurement. Chronoamperometric curves obtained at 0.497 V vs. Ag/AgCl for carbon felt electrode after 30 minute immersion in the M9 media containing 5% LB and 10mM L-Arabinose at various pHs.
(TIF)
