# Supplementary figures and images for: Anditalea andensis ANESC-ST - An Alkaliphilic Halotolerant Bacterium Capable of Electricity Generation under Alkaline-Saline Conditions
Source: PLoS One. 2015 Jul 14;10(7):e0132766. doi: 10.1371/journal.pone.0132766 (PMC4501810; doi:10.1371/journal.pone.0132766)

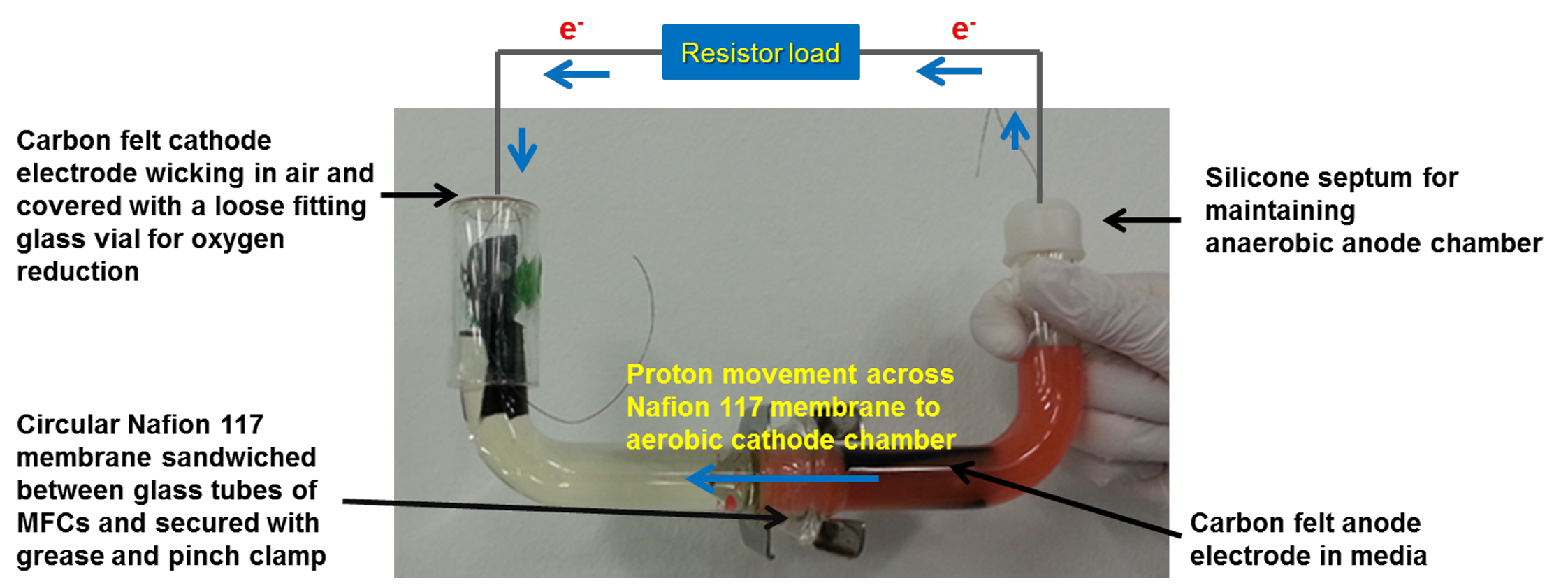

Supplement: S1 Fig — (TIF) [file pone.0132766.s001.tif]

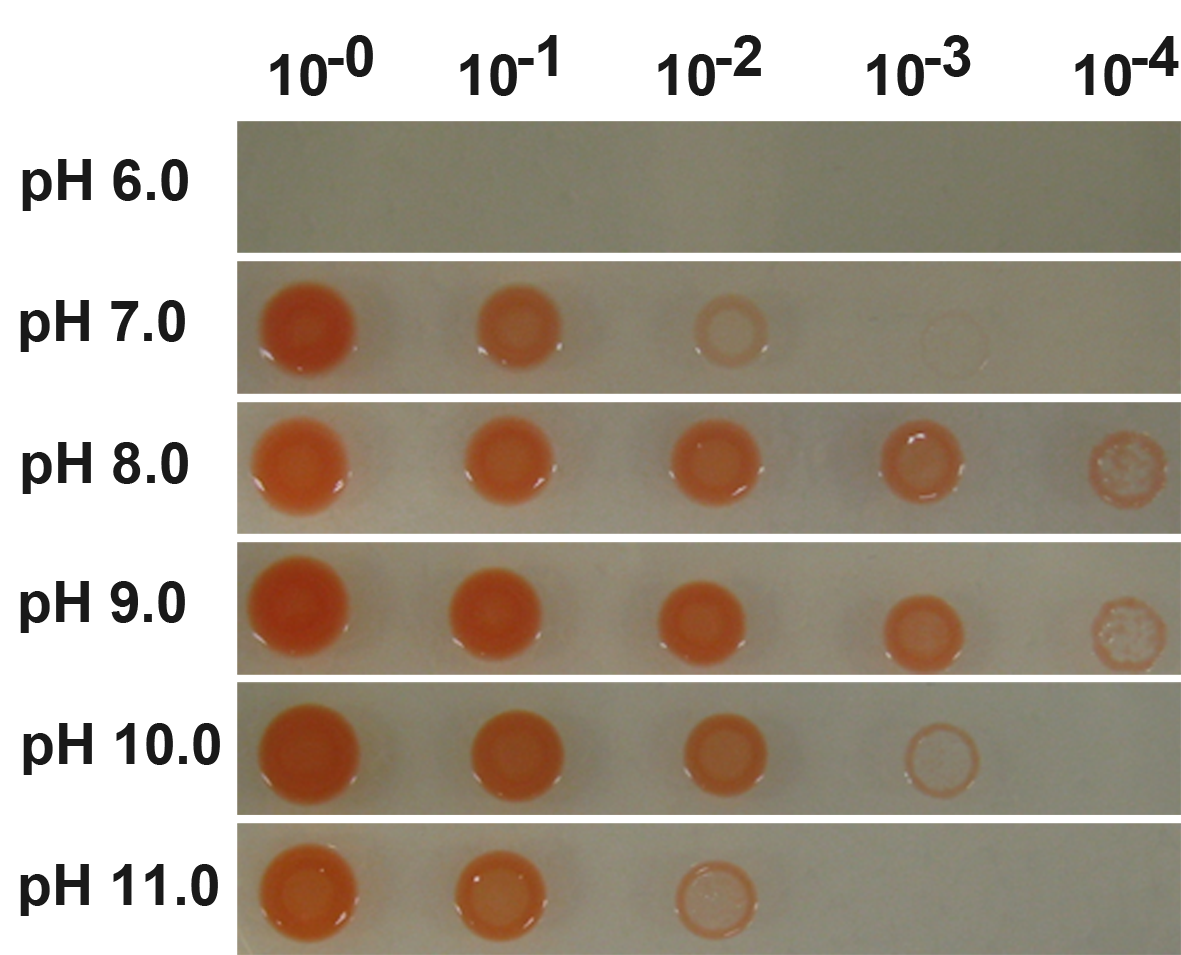

Supplement: S2 Fig — Agar plates of A. andensis ANESC-ST cultured for 48 hours under varied pH conditions with different seeding densities (Initial OD600 ≈ 0.6 for 100 to 10−4 diluted cultures). (TIF) [file pone.0132766.s002.tif]

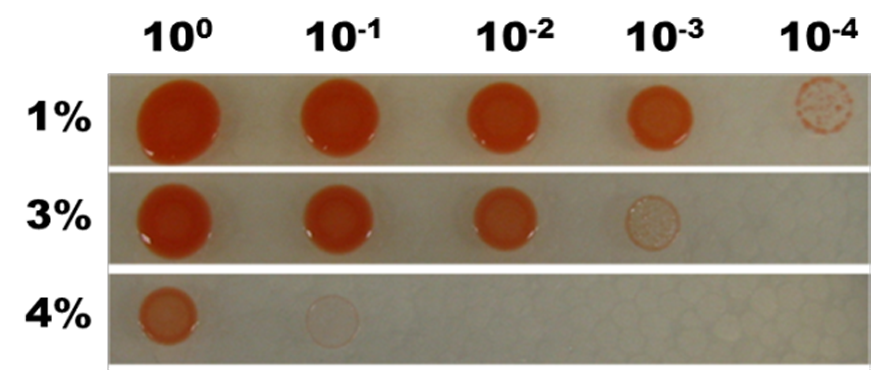

Supplement: S3 Fig — A. andensis ANESC-ST was inoculated and cultured for 48 hours in LB agar with varied NaCl concentrations (1%, 3%, and 4%) under different seeding densities (100 to 10−4 diluted cultures). (TIF) [file pone.0132766.s003.tif]

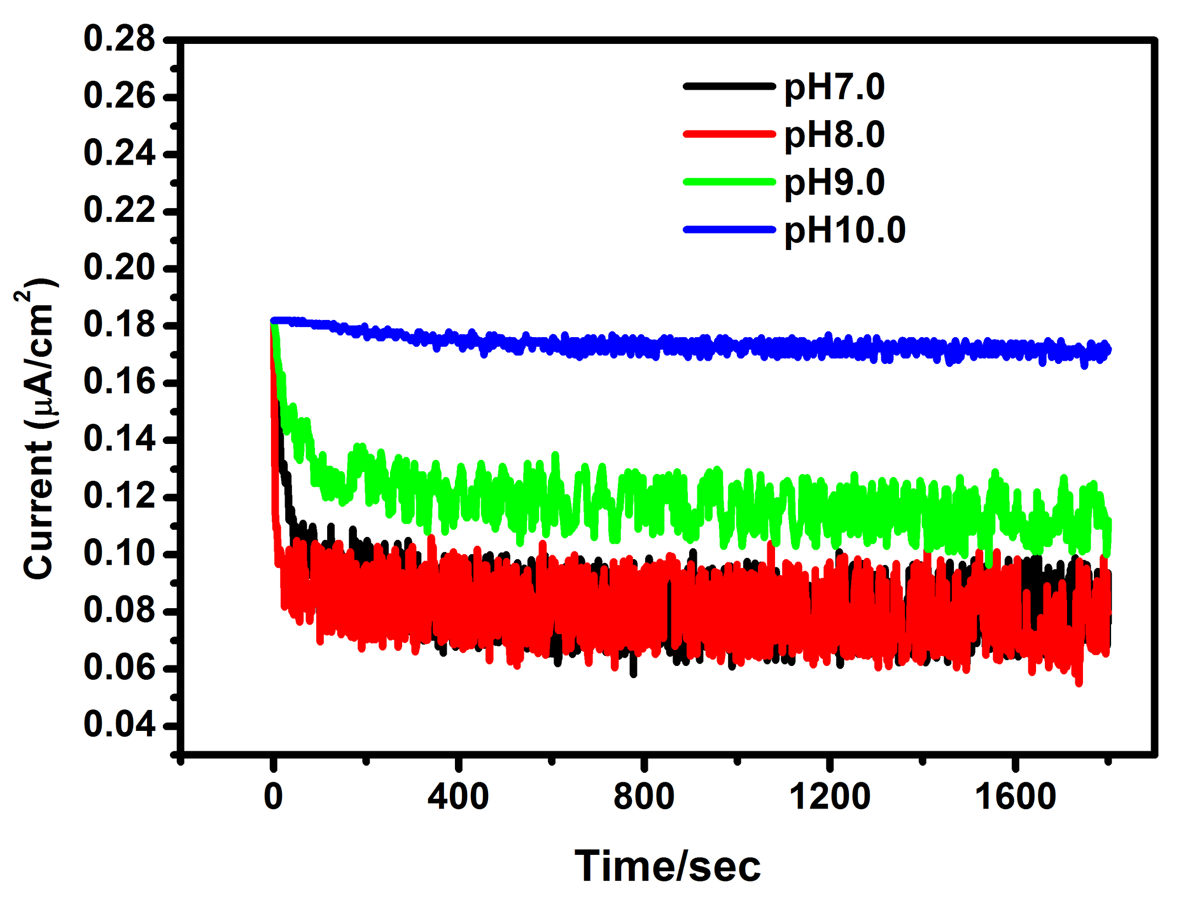

Supplement: S4 Fig — Chronoamperometric curves obtained at 0.497 V vs. Ag/AgCl for carbon felt electrode after 30 minute immersion in the M9 media containing 5% LB and 10mM L-Arabinose at various pHs. (TIF) [file pone.0132766.s004.tif]
